# Evolution and diabetic vasculopathy

**DOI:** 10.1111/jdi.13843

**Published:** 2022-06-09

**Authors:** Hiroshi Yamamoto, Yasuhiko Yamamoto

**Affiliations:** ^1^ Komatsu University Komatsu Japan; ^2^ Department of Biochemistry and Molecular Vascular Biology Kanazawa University Graduate School of Medical Sciences Kanazawa Japan

## Abstract

Evolution of blood sugar, glycation, receptor for advanced glycation end‐products and diabetic vasculopathy.

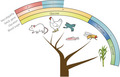

Diabetes mellitus is still an illness that leads to earlier death than in individuals who do not have this condition. The life expectancy in patients with diabetes is approximately 10 years shorter than in the non‐diabetes population. The top three causes of death in diabetes patients are malignant neoplasm, infectious disease and vascular derangement. Even before being fatal, diabetes patients often suffer from decreased quality of life due to vascular complications; for example, retinopathy can cause blindness. Nephropathy can render patients to undergo hemodialysis. In more than half of diabetes patients, signs of neuropathy are evident before symptoms become noticed. Accordingly, to know how blood vessels are impaired under diabetic conditions is important for both the extension of vital prognosis and the improvement of quality of life in diabetes patients.

As is diabetes itself, diabetic vasculopathy is a multifactorial disease that is caused by a variety of environmental and genetic factors. To screen those factors, we initially carried out an *in vitro* approach, in which pericytes and endothelial cells, the constituents of small vessels, were cultured under various chemical or physical environments. The rationale behind this approach was based on the pathological features of the early phase of diabetic retinopathy; that is, pericyte loss and focal angiogenesis. Several substances or physical conditions, such as endothelial growth factor and hypoxia, caused the increase in both pericytes and endothelial cells. Some other factors, such as high concentration of glucose, caused the decrease in both cell types. There is only one condition that consistently elicits the decrease in pericytes on one hand and the increase in endothelial cells on the other hand. It is advanced glycation end‐products (AGEs), and we thus regard it as the major environmental cause of diabetic vasculopathy. AGEs is a general term of heterogenous compounds that are yielded through non‐enzymatic glycation of macromolecules, including proteins, deoxyribonucleic acids, ribonucleic acids and lipids. The reducing carbonyl terminus of glucose can interact with the amino groups of proteins, nucleic acids and lipids, forming reversible Schiff base and Amadori rearrangement products, which further undergo irreversible chemical reactions, eventually yielding AGEs.

Our subsequent *in vivo* approach using gene‐manipulated animals showed that the major cellular device that responds to AGEs is the receptor for AGEs (RAGE). RAGE was first described in 1992 as a cell‐surface protein belonging to the immunoglobulin superfamily^1^. We found that when overexpressed in diabetic animals, the indices of retinopathy and nephropathy were aggravated^2^. On the contrary, RAGE‐null mice did not develop diabetic nephropathy^3^.

These observations suggested AGEs and RAGE could be potential molecular targets for overcoming diabetic vascular complications. Prototype chemicals used for trials against AGEs, such as aminoguanidine, which covalently binds Amadori intermediates, and AGEs breakers, which were expected to decompose pre‐existing AGEs, were likely to have stoichiometric problems, purportedly giving little or marginal effects. We found that low‐molecular weight AGEs could antagonize the action of physiologically occurring AGEs molecules^4^. This suggests that signaling ligands could possess considerable size with plural binding sites for oligomerizing RAGE molecules to transduce signals, and that rational drug design against RAGE could be possible. The amino acid residues on human RAGE protein that are essential for ligand recognition have been identified^5^.

Diabetic vasculopathy only develops in mammals, whereas diabetes‐like conditions are observed among a wide range of species. One may ask; how such diversity has emerged? One answer can be it is the result of evolution.

Sugar species that flow in the blood stream differ among vertebrates, insects and plants (Figure 1). Vertebrates, including humans, circulate glucose as blood sugar through their vascular system. Blood sugars that insects and plants use are trehalose and sucrose, respectively. Neither trehalose nor sucrose have any carbonyl group, which if present can cause glycation. *Drosophila melanogaster* mutants manifesting higher blood trehalose levels are reported, but are known to have a rather longer lifespan. Glucose consists of six carbon atoms, and, therefore, they automatically form a ring structure, because of intra‐atomic bond angles. However, when glucose transits between its two ring forms, namely, α and β anomers, it transiently gives rise to the straight‐chain structure, whose carbonyl terminus then shows a reducing, glycating activity. This is the reason why glycation takes place in vessels of vertebrates. Dr Tarui remarked in his book^6^ that diabetes might be a disease that is fated to have a risk of developing complications in vertebrates, which have glucose in their closed circulation system.

**Figure 1:**
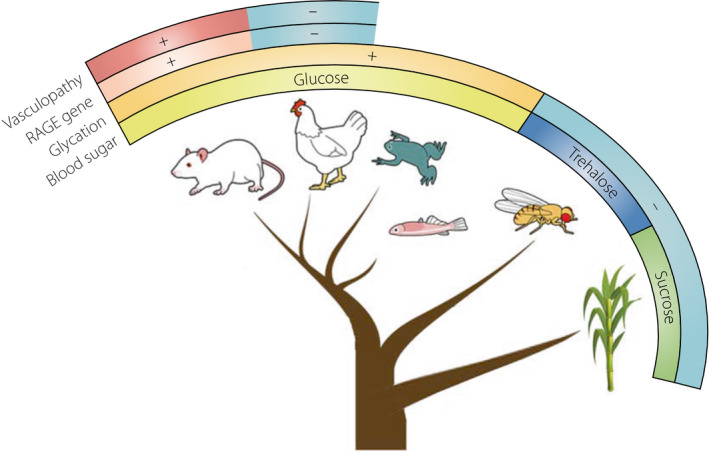
Evolution of blood sugar, glycation, receptor for advanced glycation end‐products (RAGE) gene and diabetic vasculopathy. +, presence; −, absence. Illustrations were purchased from PIXTA. [Colour figure can be viewed at wileyonlinelibrary.com]

Dr Szwergold and Dr Miller^7^ wondered why birds can live healthy lives with chronic hyperglycemia that would be fatal to humans, and analyzed the RAGE gene, *Ager*, in various mammalian and avian species. Their study showed that there are definitive homologies for the RAGE gene in humans, mice, rats, cattle, opossums and platypuses, whereas such alignments are absent from chickens, turkeys and zebra finch genomes. Accordingly, it might be reasonable to posit that the presence or absence of the RAGE gene could be a determinant of the predisposition or resistance to diabetic vasculopathy (Figure 1).

During the course of our study, we encountered an unusual observation. There were much fewer pups in the cage of RAGE‐null mice than in that of wild‐type animals. The RAGE‐null mothers did not appear to be skilled at parenting, and even committed infanticide. This seemed to be a physiologically important issue, but continued to be an enigma for a long time. Our research group, grown by 2019 to become a Japan–Russia–USA international collaboration team, finally clarified that RAGE proteins expressed on vascular cells are essential for transporting oxytocin into the brain parenchyma to elicit maternal bonding behavior^8^. Oxytocin was found to bind the V domain of RAGE protein, but at a distinct site from AGEs, neither inducing nor inhibiting intracellular signals, suggesting that targeting RAGE to overcome diabetic vasculopathy and maintaining RAGE function for parental bonding and nurturing could go together^8^.

In most likelihood, diabetes and its vasculopathy would have been beyond expectation of evolution. Our ancestors must have used glucose principally as the fuel molecule, because it yields energy very efficiently. AGEs are formed as we age, at an accelerated rate under the diabetic condition, but are almost negligible at reproductive age, the period evolution concerns. Obviously, diabetes abuses RAGE. It was probably gifted to mammals primarily as a device for parental bonding and nurturing, and numerous other socially interactive behaviors. During evolution, mammals have endowed RAGE with another benefit as an additional device for innate immunity to combat a variety of communicable and non‐communicable diseases^9^. Patients with type 2 diabetes are characterized by an undermined immune response to both natural infections and vaccination, and RAGE signaling has been implicated as a therapeutic target to improve outcomes after recent severe acute respiratory syndrome coronavirus 2 (SARS‐CoV‐2) infection^10^, ^11^. We cannot reverse the clock of evolution, although should make proper use of the yin and yang of RAGE, and other molecules that have evolved.

## DISCLOSURE

The authors declare no conflict of interest.

Approval of the research protocol: N/A.

Informed consent: N/A.

Registry and the registration no. of the study/trial: N/A.

Animal studies: N/A.
